# Regional Variations in Guideline Concordance for Women with Triple-Negative and HER2+ Breast Cancer in Nova Scotia

**DOI:** 10.3390/curroncol33060332

**Published:** 2026-06-02

**Authors:** Andrea Mayo, Hanna Stewart, Tongtong Li, Cameron Penny, Rachel Hemsworth, Ashley Drohan, Katerina Neumann, Boris Gala-Lopez, Richard T. Spence, Gregory Knapp

**Affiliations:** 1 Breast Health Research Unit, Izaak Walton Killam Health Centre, Halifax, NS B3K 6R8, Canada; andrea.mayo@iwk.nshealth.ca (A.M.); hanna.stewart@iwk.nshealth.ca (H.S.); rachel.hemsworth@iwk.nshealth.ca (R.H.); ashley.drohan@nshealth.ca (A.D.); 2 Department of Surgery, Dalhousie University, Halifax, NS B3H 2Y9, Canada; tongtong.li@nshealth.ca (T.L.); cameron.penny@dal.ca (C.P.); katerina.neumann@nshealth.ca (K.N.); boris.gala-lopez@nshealth.ca (B.G.-L.); richard.spence@nshealth.ca (R.T.S.); 3 General Surgery Research Collaborative, Dalhousie University, Halifax, NS B3H 4R2, Canada

**Keywords:** HER2, triple-negative breast cancer, neoadjuvant therapy, pathologic complete response

## Abstract

**Background**: Systematic neoadjuvant therapy (NAT) is recommended for triple-negative (TNBC) and HER2+ breast cancers when tumor size at diagnosis is ≥2 cm. This study aimed to determine the proportion of patients in Nova Scotia with non-metastatic, incident, ≥2 cm TNBC and HER2+ breast cancer who received guideline-concordant NAT, stratified by administrative zone. **Methods**: This retrospective analysis utilized data from the Nova Scotia Breast Screening Program. Adult patients (18–80 years) with an incident, non-metastatic, ≥T2 breast cancer diagnosis between 2021 and 2023 were considered theoretically eligible for NAT, defined by a wait time of ≥4 months between core biopsy and surgery. Guideline-concordant care was confirmed through chart review and compared across health system administrative zones and over time. **Results:** Of the 291 women theoretically eligible for NAT, 67.0% received it. Significant differences in NAT receipt were observed across administrative zones (Central 73.1%, Eastern 72.9%, Northern 57.4%, Western 54.2%, *p* = 0.030). **Conclusions:** This study identifies meaningful regional disparities in NAT receipt for TNBC and HER2+ breast cancer in Nova Scotia. Targeted strategies to improve guideline concordance are warranted and may lead to better patient outcomes.

## Simple Summary

Neoadjuvant chemotherapy (NAT) is beneficial for certain aggressive subtypes of breast cancers, particularly as it allows for reduced surgical intervention and improved outcomes in these patients. Some eligible patients may not be suitable for this treatment because of co-morbidities and age, but NAT should be administered to all appropriate patients according to guidelines and it should not differ by geography. In this study, we sought to describe the amount of NAT prescribed among the breast cancer population in Nova Scotia, Canada, since the release of the Canadian NAT guidelines, and understand if this differed by geographic zone. Sixty-seven percent of the eligible Nova Scotian breast cancer patients received NAT. However, differences in the receipt of NAT by health zone were found. These results indicate geographical disparities in the receipt of guideline-concordant breast cancer care and underscore the need to better understand and remedy these disparities in healthcare.

## 1. Introduction

In Canada, it is estimated that 1 in 8 women will be diagnosed with breast cancer in their lifetime [1]. Of those women, approximately 10–15% will be diagnosed with human epidermal growth factor receptor 2-positive (HER2+) breast cancer or triple-negative breast cancer (TNBC) [2,3]. These two subtypes are associated with decreased disease-free and worse overall survival compared to hormone-positive breast cancer [4,5,6]. The standard of care for early-stage (≥2 cm) TNBC and HER2+ breast cancer is to receive neoadjuvant systemic therapy (NAT) [7,8,9,10]. A large proportion of patients will experience a pathologic complete response (pCR) after NAT, while those with persistent disease derive a significant survival benefit from tailored adjuvant intervention [9,11,12,13]. In 2021, the Nova Scotia Cancer Care Program adopted and disseminated a provincial guideline for the expanded use of NAT for early-stage HER2+ and TNBC. There is a paucity of data on the pragmatic adoption of these guidelines from a province-wide perspective. The objective of this study was to quantify the proportion of patients in Nova Scotia (NS) with non-metastatic, incident, ≥2 cm TNBC and HER2+ breast cancer who received guideline-concordant therapy by administrative zone.

This article is a revised and expanded version of a paper titled ‘Abstract PS4-12-20: Regional Variation in Guideline Concordance for Women with Triple-Negative and HER2+ Breast Cancer in Nova Scotia’, which was presented at the San Antonio Breast Cancer Symposium, Texas, USA, on 11 December 2025 [14].

## 2. Materials and Methods

### 2.1. Study Design and Population

Data from the Nova Scotia Breast Screening Program’s (NSBSP) clinical information system was used to perform a retrospective cross-sectional study. The NSBSP captures diagnostic imaging and basic clinical variables of every individual with a new diagnosis of breast cancer across the province of Nova Scotia [15]. The clinical information system includes information on the breast cancer diagnosis, including immunohistochemistry, radiographic characteristics (e.g., size of the largest lesion), date and type of surgery performed, and the geographical locations of each encounter. Missing data and quality assurance were performed by cross-referencing individual patient-level data with the provincial electronic medical record. The cohort was first defined temporally, as the date of diagnostic core biopsy of invasive carcinoma between 2021 and 2023. The cohort was then further limited to women ≤ 80 years of age with a >2 cm TNBC or HER2+ breast cancer who underwent surgery within 12 months of diagnosis.

### 2.2. Outcomes and Variable Definitions

The primary outcome was the receipt of NAT, defined by a >4-month delay to surgery from diagnosis. Our secondary outcome was the incidence of pathologic complete response (pCR) after NAT, which was defined as ypT0/is ypN0 on final pathology per the AJCC guidelines. The date of diagnosis was defined as the date of the core biopsy demonstrating invasive carcinoma. Tumor size was available only from surgical pathology. Individuals eligible for NAT were determined by a tumor size < 2 cm combined with a >4-month delay to surgery from diagnosis or tumor size ≥ 2 cm with no delay. Receipt of any NAT was confirmed by review of the medical chart for all eligible patients with a ≥4-month delay from the date of diagnosis to surgery, including all patients with <2 cm tumors on final pathology.

### 2.3. Geographic and Temporal Variation

**Figure 1:**
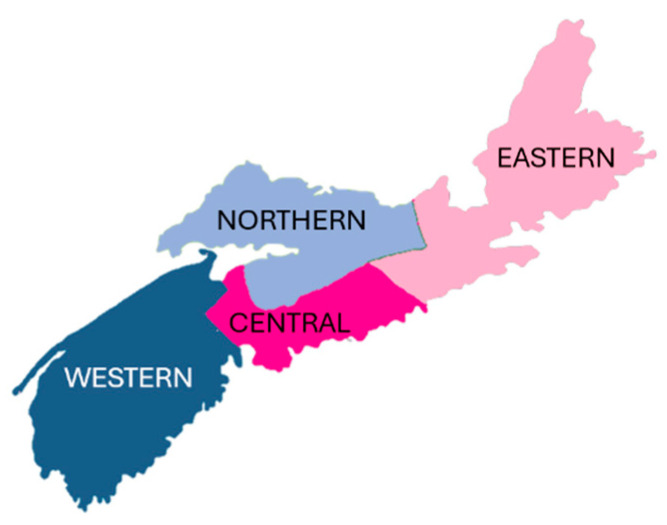
Nova Scotia Health management zones.

Differences in the receipt of NAT were examined by the administrative zone as defined by Nova Scotia Health (NSH). The zones are geographically defined areas categorized as Central, Eastern, Northern, and Western (Figure 1). Healthcare resources, planning and leadership are administered separately in each zone. An individual’s zone was classified based on their core biopsy location. Additional analyses were conducted using the year of diagnosis as the exposure variable.

### 2.4. Statistical Analysis

Demographic and diagnostic variables were summarized by zone. Median (interquartile range) for non-normally distributed data and proportions (%) were reported for continuous and categorical variables, respectively. Chi-squared tests were performed to assess heterogeneity in the proportions of patients receiving treatment across the zones. This study was based on a complete provincial cohort of individuals diagnosed with TNBC or HER2+ breast cancer in NS between 2021 and 2023. Statistical inference was not relevant in the traditional sense; however, we continued to report *p*-values from chi-squared tests as descriptive indicators of heterogeneity across zones and time rather than for formal inference. Similarly, we aimed to generalize our findings to a time period beyond this sample. All statistical analyses were performed using R Statistical Software (v4.4.2, 31 October 2024; R Core Team 2021). Ethics approval was obtained from the Nova Scotia Research Ethics Board (REB#1030580) and the Izaak Walton Killam Health Centre Research Ethics Board (REB#1027506).

## 3. Results

### 3.1. Descriptive Analysis

Of the 462 women in NS diagnosed with HER2+ or TNBC in 2021–2023, 291 women were theoretically eligible for NAT and included in this study. The median age was 59 (IQR 48–67) (Table 1). Forty-five percent (130/291) were treated for breast cancer in the Central Zone. Of the cohort, 50.5% (n = 147) were HER2+ and 49.5% (n = 144) were triple-negative, with the Central Zone having the highest proportion of HER2+ cancers (53.1%, 69/130) and the Western Zone having the highest proportion of TNBC (54.2%, 26/48) compared to the other zones. Approximately 23% (68/291) of the eligible patients had screen-detected cancers. None of these variables differed meaningfully across the zones.

### 3.2. Guideline Concordance

**Figure 2:**
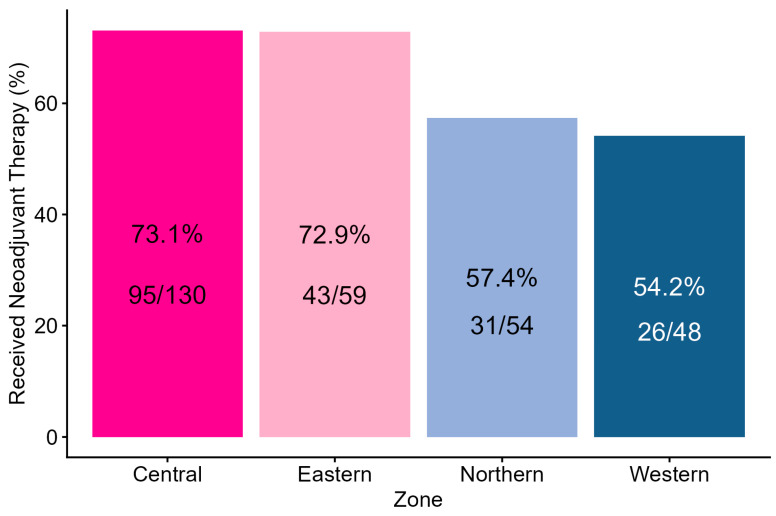
Proportion of women in Nova Scotia diagnosed with triple-negative or HER2+ breast cancer in 2021–2023 who were theoretically eligible for neoadjuvant chemotherapy (tumor size ≥ 2 cm and age < 80 years) and who received the treatment, stratified by Nova Scotia Health administrative zone.

**Figure 3:**
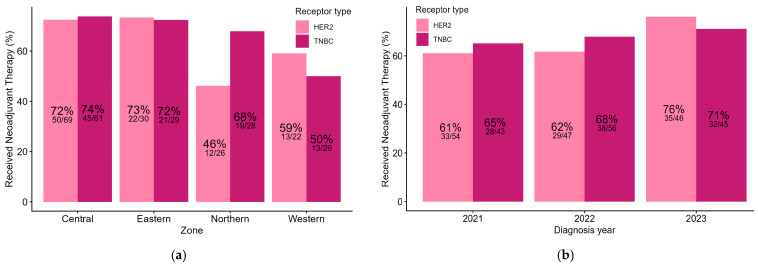
Proportion of women in Nova Scotia diagnosed with triple-negative or HER2+ breast cancer who were theoretically eligible for neoadjuvant chemotherapy (tumor size ≥ 2 cm and age < 80 years) and received neoadjuvant chemotherapy in 2021–2023. Data are characterized by tumor receptor immunohistochemistry and presented by (**a**) Nova Scotia Health zone and (**b**) diagnosis year.

Of the 291 women who were theoretically eligible for NAT (i.e., tumor sizes ≥ 2 cm and ≤80 years old), 67.0% (n = 195) received NAT. Those who received NAT were younger in median age (55 years; IQR 47–64) than those who did not receive NAT (66 years; IQR 55–72; *p* < 0.001) (Table S1). Patients in the Western and Northern Zones were less likely to receive NAT (54.2%, 26/48; and 57.4%, 31/54, respectively) compared to patients from the Central or Eastern zones (73.1%, 95/130; and 72.9%, 43/59, respectively) (Figure 2). The receipt of NAT between zones had a chi-square *p*-value of 0.030, indicating heterogeneity. Stratifying by receptor type, overall, 66.0% (97/147) and 68.1% (98/144) of HER2+ and TNBC patients received NAT, respectively, with the Northern and Western Zones having the lowest proportions of NAT receipt (Figure 3a).

**Figure 4:**
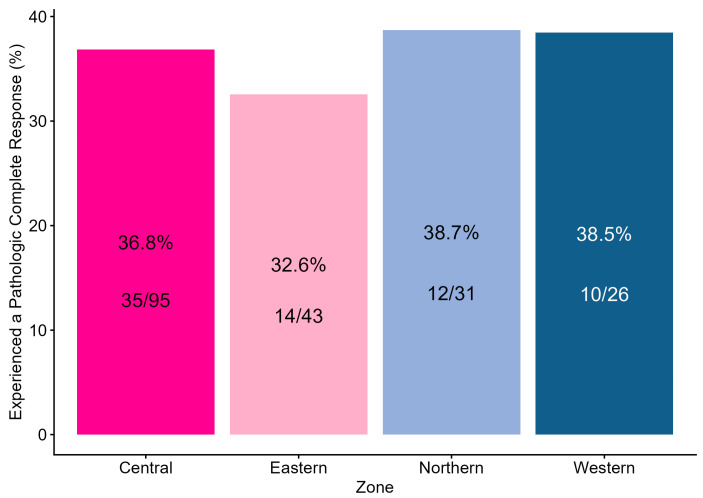
Proportion of women in Nova Scotia diagnosed with triple-negative or HER2+ breast cancer in 2021–2023 who experienced a pathologic complete response following neoadjuvant chemotherapy, stratified by Nova Scotia Health management zone.

The proportion of NAT receipt increased by year, with 2023 having the highest proportion of NAT receipt (73.6%, 67/91) compared to 2021 and 2022 (62.9%, 61/97; and 65.1%, 67/103, respectively). When stratified by receptor type, HER2+ breast cancer had an absolute 15% increase in NAT receipt from 2021 to 2023, whereas TNBC saw an absolute increase of 6% between these years (Figure 3b). Among individuals who received NAT (n = 195), 36.4% (n = 71) achieved a pCR. Rates of pCR were consistent across the zones (32.6– 38.7%, *p* = 0.940) (Figure 4).

## 4. Discussion

The province of NS has the highest breast cancer-specific mortality in Canada [1,16]. Receipt of guideline-concordant oncology care ensures that cancer patients receive evidence-based treatments, which can improve cancer-free survival [17,18] and quality of life by improving tumor control and surgical options [18,19,20]. In NS, the majority of women with HER2+ and TNBC receive guideline-concordant NAT, including those with early-stage disease. However, access is not consistent across the province and appears to vary by the administrative zone of diagnosis. Patients in the Central Zone were 19% more likely to receive guideline-concordant care compared to patients in the Western Zone (54.2%). The Central Zone corresponds to the province’s largest urban area (i.e., Halifax) and one of two zones with multidisciplinary cancer centers (i.e., Halifax and Sydney). Despite geographic differences in the proportion of women receiving NAT, similar response rates were noted among those patients who receive guideline-concordant care.

While relatively compact in terms of geographic size, previous research regarding healthcare access and outcomes in NS has demonstrated important differences on the basis of rurality [21,22]. Indeed, geographic disparities in breast cancer care have also been documented in other provinces despite the publicly funded nature of the Canadian healthcare system. Much of the literature on this topic has focused on regional variation in the utilization of BCS versus mastectomy and the subsequent need for adjuvant radiotherapy [23,24,25,26,27,28,29]. The provision of these two services is intimately related, and geographic inequities in the availability of radiotherapy have been associated with reduced rates of BCS in regions where patients must travel longer distances to receive adjuvant radiotherapy [24,25]. Given the comparable survival and superior quality of life that has been documented following BCS relative to mastectomy, variations in surgical modality—as a function of geographic location—merit careful consideration and scrutiny in a publicly funded system [30,31,32]. Interestingly, rurality may not influence the receipt of adjuvant chemotherapy in Canada to the same extent as the receipt of adjuvant radiotherapy in the setting of breast cancer. In a study examining geographic treatment variation among breast cancer patients in British Columbia, receiving care in a rural health authority was associated with decreased odds of undergoing BCS, but was not associated with the receipt of chemotherapy [28]. This may be, in part, due to important structural differences in the provincial organization of chemotherapy and radiotherapy services, with distributed oncology clinics in rural health authorities that provide systemic therapy outside of urban centers [28]. The database utilized for this study only captured clinical information pertaining to breast cancer screening and diagnosis; therefore, treatment differences by healthcare zone could not be captured.

To our knowledge, only one additional study has explored geographic variation in the receipt of guideline-concordant NAT for HER2+ and TNBC in Canada [33]. In a population-based cohort study of women in Ontario with stage I–III HER2+ or TNBC, distance from the nearest cancer center was not associated with the likelihood of referral to medical oncology for consideration of NAT. However, residing 10–25 km from the nearest cancer center was associated with a reduced likelihood of receiving NAT relative to those who resided closer (<5 km) to the nearest cancer center. Notably, distances greater than 25 km were not negatively associated with the receipt of guideline-concordant NAT [33]. The follow-up periods in the current study and the Ontario-based study (2021–2023 versus 2012–2020, respectively) differ importantly relative to the timing of publication of the majority of evidence that prompted guideline changes surrounding NAT in this patient population. Thus, it is not possible to directly compare the overall proportion of patients receiving guideline-concordant NAT in the two studies (67.0% in Nova Scotia versus 23.9% in Ontario) [33]. The finding by Yee et al. that distance from the nearest cancer center had a deleterious effect on the receipt of NAT at intermediate distances, but not on consultation with medical oncology at any distance, is worth noting. Once contact with medical oncology is made, there may be important geographic variation in system- and patient-level factors that influence the receipt of NAT [33]. As demonstrated by patterns of adjuvant systemic therapy use among breast cancer patients in British Columbia, the regionalization of systemic therapy through satellite clinics could provide a means of increasing the uptake of NAT, provided appropriate initial referrals to medical oncology are made [28].

In Nova Scotia, the Central Zone largely comprises the city of Halifax and the associated municipality. This is the most urban environment in the province and includes the largest tertiary care facility and cancer center in Atlantic Canada. There is a satellite cancer center in Sydney, the province’s second-largest urban center, which is located in the Eastern Zone [34]. The finding that patients are more likely to receive guideline-concordant care in these two administrative zones may be a function of the heterogeneous access to centralized and multidisciplinary care across the province. For instance, in accordance with the national Canadian guidelines [35], the use of a multidisciplinary team in discussions regarding the initiation of NAT is more feasible in larger centers, where well-coordinated, centralized breast cancer care pathways are more likely to co-exist. Patients diagnosed and treated in administrative zones without a cancer center (i.e., Western and Northern Zones) are more likely to receive their primary consultation and initial cancer management through an individual surgeon’s office. In the Central zone, patients are flagged for consideration of neoadjuvant therapy at the time of referral through the Cancer Care program. This referral system may have contributed to the higher rate of NAT receipt observed in the Central Zone. Implementing a similar system in the other health zones, where current referrals to medical oncology rely on surgeon knowledge and practice patterns, may increase the uptake of NAT in peripheral centers. The timing of referral and choice of surgical provider is at the discretion of the primary care provider who receives the core biopsy results (i.e., breast cancer diagnosis). The presence of a cancer center and a centralized multidisciplinary team may improve the likelihood of receiving NAT, as comprehensive cancer centers have been shown to have higher adherence to quality indicators and guideline adherence [36,37]. In the current study, receipt of NAT among eligible patients was highest in administrative zones with a dedicated cancer center and generally increased by year for the provincial cohort. Taken together, these findings may suggest that provider knowledge could be an important target for intervention to increase the uptake of NAT among HER2+ and TNBC patients in Nova Scotia.

The strength of this study is that, despite the small sample size, it represents a near-complete provincial cohort. Every individual diagnosed with breast cancer within our time period should be captured in the breast imaging clinical information pathway used to identify cases. This limits the influence of any sampling variability on the results. However, this study is not without limitations. We did not consider any influence confounding may have on the relationship between the zones and our outcome variables. Unmeasured and unadjusted confounders, such as age or income, might contribute to any observed differences. No causality should be assumed from this study. This study is exploratory in nature, given that the patients deemed eligible for receipt of the intervention and those categorized as having received the intervention were based only on tumor size and time from diagnosis to surgery, respectively. Patients with an appropriate TNM stage for NAT may have declined treatment or had other medical contraindications. However, we see no reason to suggest that the proportion of such patients would differ between administrative zones. While uncommon, there may be individuals who received some amount of NAT (i.e., less than a full chemotherapy regimen) who were not captured as having NAT if they had a duration of less than 4 months between diagnosis and surgery and a tumor size smaller than 2 cm on final surgical pathology. However, we see no reason that any variations in time from diagnosis to surgery would be unevenly distributed across the province. The findings from this study could be compared with provincial pharmacy or billing data to better capture definitive neoadjuvant treatments.

It is possible that there is some misclassification within this study. While a patient’s zone was defined as the core biopsy procedure location, it is possible that their zone of treatment differed. A sensitivity analysis using the surgical zone instead of core biopsy location did not meaningfully alter the results. We continued to see the highest proportion of NAT receipt in the Central and Eastern Zones and the lowest proportions in the Northern and Western Zones (Table S2). The increase in Central Zone patients (140 vs. 130) under surgical zone classification is consistent with selective referral patterns of complex breast cancer diagnoses to the tertiary or high-volume clinics in the central zone for operative management. It is likely zone classification on surgical location would more often misattribute their main zone of care and obscure true differences in NAT receipt across the regions compared to core biopsy location. Similarly, eligibility for NAT in this study utilized pathologic tumor size. Misclassification of eligibility may be possible where a patient was <2 cm clinically and ineligible for NAT but ≥2 cm by surgical pathology. Although it is impossible to calculate the magnitude of eligibility misclassification from the available data in this study, future research should consider a more robust approach to identifying eligible patients. Finally, it is important to note that the observed differences in the proportion of NAT receipt may also reflect the timeframe in which the data was collected. The years of data collection for this study (2021–2023) are the years following the recognition of the COVID-19 pandemic in early 2020 and are associated with abnormal practice patterns across the healthcare system [38,39]. Previously published research has shown that care during COVID-19 differed by rurality [40,41]. This might explain why we found an uptick in guideline concordance for HER2+ or triple-negative breast cancer patients between 2021 and 2023, as the healthcare system returned to baseline.

## 5. Conclusions

There may be variability in receipt of guideline-concordant care for HER2+ and TNBC in Nova Scotia based on the location of diagnosis and primary treatment. By highlighting regional variations, this study underscores the need for prospective data on key quality indicators and the role for targeted data-driven healthcare policies and interventions to address system factors associated with poor outcomes. Future research should explore how these factors contribute to treatment inequities and identify strategies to mitigate the impact of access disparities on breast cancer outcomes.

## Figures and Tables

**Table 1:** Characteristics of women diagnosed with triple-negative or HER2+ breast cancer in Nova Scotia between 2021 and 2023 by Nova Scotia Health management zones (n = 291). Reported as n (%) unless otherwise specified.

Characteristics	Total (n = 291)	Central (n = 130)	Eastern (n = 59)	Northern (n = 54)	Western (n = 48)
**Demographic variables**					
Age at diagnosis (years), median (IQR)	59.0 (48.0–67.0)	59.0 (48.2–66.8)	60.0 (51.5–68.0)	58.5 (48.2–67.8)	58.5 (45.8–64.2)
**Diagnostic variables**					
Screen detected					
Yes	68 (23.37)	29 (22.31)	13 (22.03)	15 (27.78)	11 (22.92)
No	223 (76.63)	101 (77.69)	46 (77.97)	39 (72.22)	37 (77.08)
Receptor type					
HER2+	147 (50.52)	69 (53.08)	30 (50.85)	26 (48.15)	22 (45.83)
Triple-negative	144 (49.48)	61 (46.92)	29 (49.15)	28 (51.85)	26 (54.17)

Abbreviations: IQR, interquartile range; HER2+, human epidermal growth factor receptor 2 positive.

## Data Availability

The datasets presented in this article are not readily available because of privacy and ethical considerations in line with Chapter 5 of the 2022 Tri-Council Policy Statement: Ethical Conduct for Research Involving Humans, which pertains to the use of identifiers in this study. Data were obtained from the Nova Scotia Breast Screening Program (NSBSP), and requests to access the datasets should be directed to the NSBSP.

## Supplementary Materials

The following supporting information can be downloaded at https://www.mdpi.com/article/10.3390/curroncol33060332/s1, Table S1: Characteristics of women diagnosed with triple-negative or HER2+ breast cancer in Nova Scotia between 2021 and 2023 by receipt of neoadjuvant chemotherapy status; Table S2: Neoadjuvant chemotherapy receipt and pathologic complete response rates of women diagnosed with triple-negative or HER2+ breast cancer in Nova Scotia between 2021 and 2023, categorized by Nova Scotia Health management zone and surgical location.

## Abbreviations

The following abbreviations are used in this manuscript:

BCS	Breast-conserving surgery
HER2	Human epidermal growth factor receptor 2
NAT	Neoadjuvant chemotherapy
NS	Nova Scotia
NSBSP	Nova Scotia Breast Screening Program
NSH	Nova Scotia Health
pCR	Pathologic complete response
TNBC	Triple-negative breast cancer
