# Supplementary material for: Regional Variations in Guideline Concordance for Women with Triple-Negative and HER2+ Breast Cancer in Nova Scotia
Source: Curr Oncol. 2026 Jun 2;33(6):332. doi: 10.3390/curroncol33060332 (PMC13298986; doi:10.3390/curroncol33060332)
Supplement: Supplementary file 1 [file curroncol-33-00332-s001.zip › curroncol-4252361-supplementary.pdf]

Table S1. Characteristics of women diagnosed with triple-negative or HER2+ breast cancer in Nova Scotia between 2021 and 2023 by receipt of neoadjuvant chemotherapy status (n = 291). Reported as n (%) unless otherwise specified.

Characteristics	Neoadjuvant Chemotherapy (n = 195)	No Neoadjuvant Chemotherapy (n = 96)	p-value
**Demographic variables**			
Age at diagnosis (years), median (IQR)	55.0 (47.0-64.0)	66.0 (55.0-72.0)	<0.001*
**Diagnostic variables**			
Screen detected			0.178**
Yes	41 (21.0)	27 (28.1)	
No	154 (79.0)	69 (71.9)	
Receptor type			0.707**
HER2+	97 (49.7)	50 (52.1)	
Triple-negative	98 (50.3)	46 (47.9)	
Surgery type			
BCS	86 (44.1)	49 (51.0)	0.264**
Mastectomy	109 (55.9)	47 (49.0)	

*Kruskal-Wallis test. **Chi-squared test. Abbreviations: IQR, Interquartile range; HER2+, Human epidermal growth factor receptor 2 positive; BCS, Breast conserving surgery.

Table S2. Neoadjuvant chemotherapy receipt and pathologic complete response rates of women diagnosed with triple-negative or HER2+ breast cancer in Nova Scotia between 2021 and 2023 by Nova Scotia Health management zones categorized by surgical location (n = 291). Reported as n (%).

Characteristics	Total (n = 291)	Central (n = 140)	Eastern (n = 54)	Northern (n = 51)	Western (n = 46)
**Neoadjuvant Therapy**	195 (67.0)	100 (71.4)	38 (70.4)	32 (62.8)	25 (54.4)
HER2+	97 (66.0)	54 (72.0)	19 (67.9)	13 (54.2)	11 (55.0)
TNBC	98 (68.1)	46 (70.8)	19 (73.1)	19 (70.4)	14 (53.9)
**Pathologic Complete Response (yes)**	71 (36.4)	36 (36.0)	12 (31.6)	14 (43.8)	9 (36.0)
HER2+	42 (43.3)	22 (40.7)	8 (42.1)	8 (61.5)	4 (36.4)
TNBC	29 (29.59)	14 (30.4)	4 (21.1)	6 (31.6)	5 (35.71)

Abbreviations: HER2+, Human epidermal growth factor receptor 2 positive; TNBC, triple-negative breast cancer.
